# Gene expression profiling of whole blood samples following marathon running in non-elite athletes

**DOI:** 10.5114/biolsport.2026.158303

**Published:** 2026-01-23

**Authors:** Pol Ezquerra-Condeminas, Laura Martin-Fernandez, Antonio Cardenas, Oriol Sibila, Nina Borràs, Francisco Vidal, Alexandre Perera-Lluna, José Manuel Soria

**Affiliations:** 1 Unit of Genomics of Complex Disease, Research Institute of Sant Pau Hospital (IIB Sant Pau), Spain; 2 B2SLab, Institute for Research and Innovation in Health (IRIS), Universitat Politècnica de Catalunya-BarcelonaTech, Barcelona, Spain; 3 Centre for Biomedical Network Research on Rare Diseases (CIBERER), Instituto de Salud Carlos III, Madrid, Spain; 4 Congenital Coagulopathies Laboratory, Blood and Tissue Bank, Barcelona, Spain; 5 Transfusional Medicine, Vall d’Hebron Research Institute, Universitat Autònoma de Barcelona (VHIR-UAB), Barcelona, Spain; 6 Respiratory Department, Hospital Clínic, IDIBAPS, CIBERES, University of Barcelona, Barcelona, Spain; 7 Centre for Biomedical Network Research of Cardiovascular Diseases (CIBERCV), Instituto Carlos III (ISCIII), Madrid, Spain; 8 Centre for Biomedical Network Research of Bioengineering, Biomaterials and Nanomedicine, Instituto Carlos III (ISCIII), Madrid, Spain

**Keywords:** RNA-seq, Endurance exercise, Transcriptomics, Gene expression profiling, Marathon running, Exercise-induced gene regulation

## Abstract

Endurance exercise exerts profound physiological effects on non-elite athletes, but the underlying molecular mechanisms remain incompletely understood. This study investigated transcriptomic changes induced by running a marathon and their progression during recovery. Blood samples were collected from 60 non-elite athletes (42 men, 18 women) at three time points: baseline (START), immediately after the marathon (FINISH), and 24 hours post-race (24REST). Differential gene expression analyses, along with Gene Ontology (GO) and KEGG pathway enrichment, were performed for three comparisons: C1 (START vs FINISH), C2 (FINISH vs 24REST), and C3 (START vs 24REST). The analysis identified 9,874 differentially expressed genes (DEGs) in C1, indicating widespread gene expression changes following the marathon. GO and KEGG analyses highlighted significant enrichments in biological processes such as immune function, oxidative stress response, and lipid metabolism. At 24REST, gene expression had not fully returned to baseline, with 279 DEGs observed in C3. These genes were predominantly associated with mitochondrial function and energy production pathways, suggesting differences in mitochondrial and energy associated gene expression compared with baseline. Clustering analyses identified two clusters of recovered genes and three clusters of differentially expressed genes at 24REST, reflecting distinct temporal expression trajectories. These findings underscore the substantial impact of endurance exercise on gene expression in non-elite athletes, providing a foundation for understanding transcriptomic regulation in non-elite athletes and may inform strategies to optimize recovery. Future research is necessary to explore the long-term physiological and health implications of these transcriptional changes.

## INTRODUCTION

Previous studies in sports science suggest that regular exercise is beneficial for our health, for example, it reduces the chances of developing heart disease [1–4].

Nevertheless, the consequences of performing exhausting endurance activities remain unclear. For example, a study looking at *Tour de France* participants found a significantly lower rate of cardiovascular events and longer life expectancy compared to agematched, general French men population [5]. Conversely, subjecting the body to the extremes of rigorous training, such as marathon running, might lead to adverse effects [6, 7]. The reasons behind these divergent outcomes may lie in various factors that influence how our bodies respond to intense physical exertion. It has been posited that age and the conditions under which training occurs could account for these differences [8].

To better understand what happens in the human body, the present study analyzed changes in the gene expression of subjects after they had run a marathon. Previous work focusing on gene expression during an ultra-marathon reported a significant impact on the immune and inflammatory systems [9]. Furthermore, enriched pathways were linked to protein synthesis repression, an altered immune system, and infectious disease-related mechanisms. Some studies suggest that strenuous exercise can induce immune system dysfunction and increase the risk of infection while endurance activities could also have a direct impact on fatty acid metabolism [10, 11]. High-intensity training may increase the prevalence and severity of coronary atherosclerosis and modify insulin resistance in humans [12, 13].

Consequently, research into the impact of endurance sports on health, both at a professional or amateur level, has grown recently. Therefore, the aim of this study was to assess the effect of a demanding intense physical effort on gene expression using RNA extracted from whole blood samples, with a particular focus on understanding the mechanisms involved in recovery to mitigate any negative effects. This was done by identifying genes with significantly different expression levels after the subjects had run a marathon; then studying the genes’ biological implications through pathway and gene ontology term enrichment analyses and assessing their significance during the race; and finally defining their expression recovery levels 24 hours after the strenuous exercise.

## MATERIALS AND METHODS

### Study Design

A total of 10 mL of whole blood was collected from 78 non-elite athletes at three distinct time points surrounding their participation in the 2016 Barcelona Marathon, a 42-kilometer endurance event. After comprehensive quality control (QC) assessment, which considered RNA concentration, final library concentration, and sequencing performance metrics (see Supplementary Table 1), 18 participants were excluded due to poor RNA quality or incomplete extractions. The final dataset comprised 60 individuals (42 men and 18 women), with participant characteristics summarized in Table 1. The male group showed a mean height of 178.9 ± 5.5 cm, weight of 75.6 ± 7.3 kg, and finishing time of 205.8 ± 25.3 minutes, while the female group presented 165.5 ± 6.2 cm, 57.1 ± 7.0 kg, and 226.5 ± 19.0 minutes, respectively. The final dataset included 180 samples (three per participant) encompassing 14,554 genes annotated with Ensembl IDs.

**TABLE 1:** Participant characteristics by sex. This table summarizes the demographic and performance characteristics of the study participants stratified by sex. Variables include Sex, Age group (years), Height (cm, mean ± SD), Weight (kg, mean ± SD), and Finishing Time (minutes, mean ± SD). Age groups are presented with the number of participants and the corresponding percentage of the total within each sex.

Sex	Age group (years)	Height (cm)	Weight (kg)	Finishing Time (min)
Male	20–25 (3, 2.3%); 25–30 (9, 7%); 30–35 (32, 25%); 35–40 (27, 21.1%); 40–45 (42, 32.8%); 45–50 (12, 9.4%); 50–55 (3, 2.3%)	178.9 (5.5)	75.6 (7.3)	205.8 (25.3)

Female	25–30 (6, 12%); 30–35 (9, 18%); 35–40 (6, 12%); 40–45 (23, 46%); 45–50 (3, 6%); 50–55 (3, 6%)	155.5 (39.5)	57.1 (7.0)	226.5 (19.0)

**FIG. 1:**
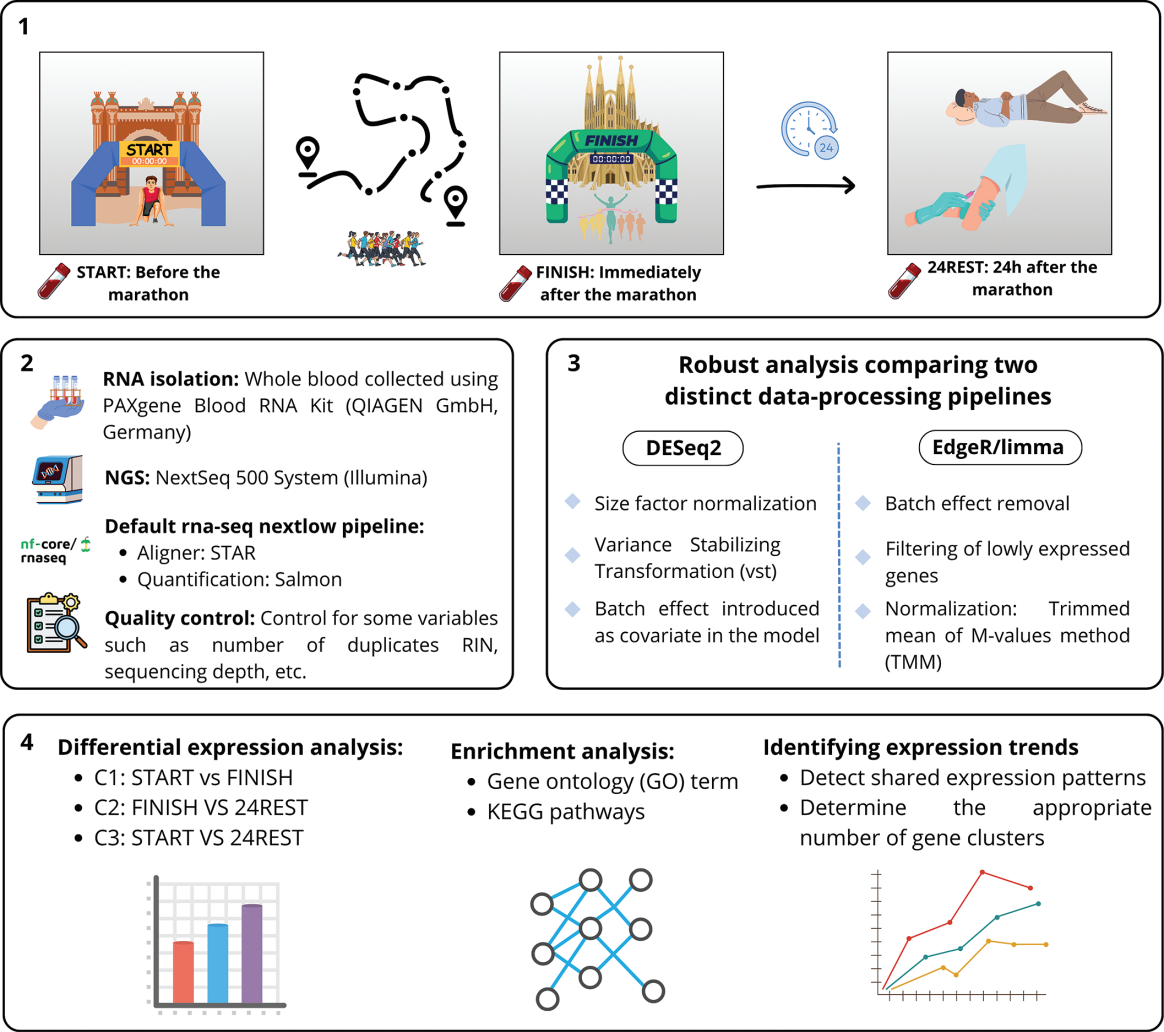
Experimental design and analysis workflow. (1) Blood samples were collected from participants at three time points: START, FINISH, and 24 hours after the marathon. (2) RNA was extracted from the samples and subjected to high-throughput sequencing. (3) Differential gene expression analyses were performed in parallel using edgeR and DESeq2. (4) Significantly regulated genes were subsequently analyzed for functional enrichment using Gene Ontology (GO) terms and KEGG pathways. Finally, genes exhibiting similar expression patterns across the time points were clustered to identify groups of co-regulated genes.

Blood samples were collected at the following time points: immediately prior to the race (START), immediately after the race (FINISH), and 24 hours post-race (24REST). These time points enabled three comparative analyses: C1, which compared FINISH to START levels; C2, which examined FINISH against 24REST levels; and C3, which assessed 24REST relative to START levels (Figure 1).

The study was conducted in accordance with the Declaration of Helsinki and was approved by the Ethics Committee at Hospital de la Santa Creu i Sant Pau (Barcelona). All participants provided written informed consent. To ensure compliance with data protection regulations, all data was managed under the framework of the General Data Protection Regulation (GDPR), safeguarding participants’ rights regarding the processing and free movement of personal data.

### RNA Extraction and Sample Preparation

Whole blood samples were collected in PAXgene Blood RNA Tubes (QIAGEN GmbH, Germany) following the manufacturer’s recommendations. Intracellular RNA was isolated using the QIAsymphony PAXgene Blood RNA Kit (QIAGEN GmbH), and total RNA was stored at -80°C until further use.

Blood samples from the FINISH time point were collected in a pavilion near the marathon finish line, equipped for blood extractions, while START and 24REST samples were obtained at Hospital de la Santa Creu i Sant Pau. All samples were sent to Catalonia’s Blood and Tissue Bank (Banc de Sang i Teixits, BST) for RNA extraction and sequencing. Globin mRNA was depleted using the GLOBINclear Kit (Invitrogen, Thermo Fisher Scientific Inc., Massachusetts, USA). RNA libraries were prepared using the Illumina Stranded mRNA Prep Kit (Illumina, San Diego, CA) according to the manufacturer’s protocol and sequenced on a NextSeq500 system (Illumina).

### Gene expression quantification and linear model fitting approaches

Sequencing was performed using a 150-cycle (2 × 74 bp) paired-end read kit. Reads were aligned to the GRCh38 reference genome with the STAR (v.2.7.10a) algorithm using default parameters, and gene quantification was conducted with Salmon (v.1.10). Gene annotation followed Ensembl guidelines.

Gene expression data were processed using two complementary analytical pipelines: the first combining the EdgeR (v4.0.16) and limma (v3.58.1) packages, and the second employing DESeq2 (1.44.0) [14, 15, 16]. For the EdgeR/limma approach, lowly expressed genes with fewer than five counts were excluded to enhance reliability and avoid lowly expressed bias. The countsper-million matrix was normalized using the weighted trimmed mean of M-values (TMM) method implemented in EdgeR, which adjusts for library size differences. Batch effects were subsequently removed using the ***removeBatchEffect*** function from limma.

For the DESeq2 pipeline, the default normalization procedure was applied, in which counts are divided by sample-specific size factors determined by the median ratio of gene counts relative to the geometric mean per gene. In this approach, batch effect correction was not performed as a preprocessing step but was instead included as a covariate in the model. Following normalization, a multidimensional scaling (MDS) analysis was performed to examine sample distribution and identify potential covariates or clustering patterns for consideration in subsequent analyses.

The primary objective of the analysis was to compare gene expression levels across three time points using the pairwise contrasts: C1 (FINISH − START), C2 (24REST − FINISH), and C3(START − 24REST). A design matrix was constructed for each analytical pipeline to incorporate covariates that could potentially bias the model. For the limma/edgeR pipeline, the design matrix included group (Vg), sex (Vs), finishing time (Vf), and age (Va), with the intercept omitted to enable direct estimation of relative changes across all time points without dependence on a reference level:


design matrix(limma/edgeR)=O+V g+V s+Vf+Va (1)

Individual variability was not included as a fixed effect. Instead, we applied the ***duplicateCorrelation*** function prior to model fitting to estimate and account for the correlation among repeated measures from the same subject.

For the DESeq2 pipeline, the same covariates were included, along with the batch effect variable (*Vbe*). In this case, the intercept was retained, as DESeq2 does not require its removal:


design matrix(DESeq2)=V g+V s+Vf+Va+Vbe (2)

Results from both analyses were compared to validate the robustness of DEG detection and confirm reproducibility of the transcriptomic patterns across time points.

### Differential gene expression analysis (DEG)

Differential expression analysis was performed for all three pairwise comparisons. In the EdgeR/limma pipeline, the ***lmFit*** function from the sva (v.3.52.0) package was used to fit a linear model to each gene. Based on these linear models, the ***eBayes*** function was applied to compute moderated *t*-statistics, moderated *F*-statistics, and logodds of differential expression through empirical Bayes moderation of the standard errors toward a common value. This approach enabled the assessment of quantitative differences in gene expression levels between experimental groups.

For the DESeq2 pipeline, the ***DESeq*** function was used to identify differentially expressed genes. All *p*-values were adjusted using the Bonferroni multiple comparison correction method, a conservative approach that minimizes false-positive results [17]. This correction was applied consistently across all analyses conducted in this study.

### Likelihood Ratio Test (LRT) analysis in DESeq2

In addition to pairwise comparisons, a Likelihood Ratio Test (LRT) was performed to identify genes exhibiting expression changes across the different conditions. The interaction between gene expression variation and the variables *sex* and marathon finishing time were also evaluated to determine whether specific genes exhibited differential expression patterns across groups as a function of these covariates. For this purpose, two additional LRT models were fitted in DESeq2: one testing the interaction between the different groups and marathon finishing time, and another testing the interaction between groups and gender, while adjusting for the other covariables in both cases.

Furthermore, significant DEGs identified across the different groups were clustered using a *k*-means algorithm to detect groups of genes with similar expression patterns [18]. The optimal number of clusters was determined using the elbow method [19]. Beyond general clustering, a subset analysis was conducted by intersecting the LRT results with cluster C3 to assess whether genes not recovered 24 hours after the marathon displayed distinct expression dynamics.

Finally, several visualizations, including hierarchical clustering, were generated using the gplots (v3.5.1) package [20].

### Functional Enrichment Analysis: Gene Ontology and KEGG Pathways

Gene Ontology (GO) enrichment analysis was performed using the GOstats (v2.68.0) package to identify both overrepresented and underrepresented GO terms [21]. Significant genes obtained from the linear models described in the previous section (i.e., genes with adjusted *p*-values < 0.05) were used as input for the GO analysis of each comparison group (C1, C2, and C3). A conditional hypergeometric test was applied to evaluate relationships among GO terms. Using a subclass of *HyperGParams*, the package computed hypergeometric *p*-values to assess the over- or underrepresentation of each GO term within the specified gene set.

Results from GOstats were filtered based on three main parameters: (i) the total number of genes included in each GO term (size), (ii) the number of observed genes from the input set (count), and (iii) the probability of observing these genes by chance (odds ratio). The filtered results were visualized using the GOplot (v1.0.2) package [22].

For the KEGG pathway analysis, the same methodology described above was applied. The hypergeometric test was performed using the signatureSearch (v1.16.0) package [23], specifically employing the ***enrichKEGG2*** function, which generates KEGG pathway enrichment results from a provided vector of gene identifiers.

## RESULTS

### Exploring covariates using multidimensional scaling analysis

**FIG. 2:**
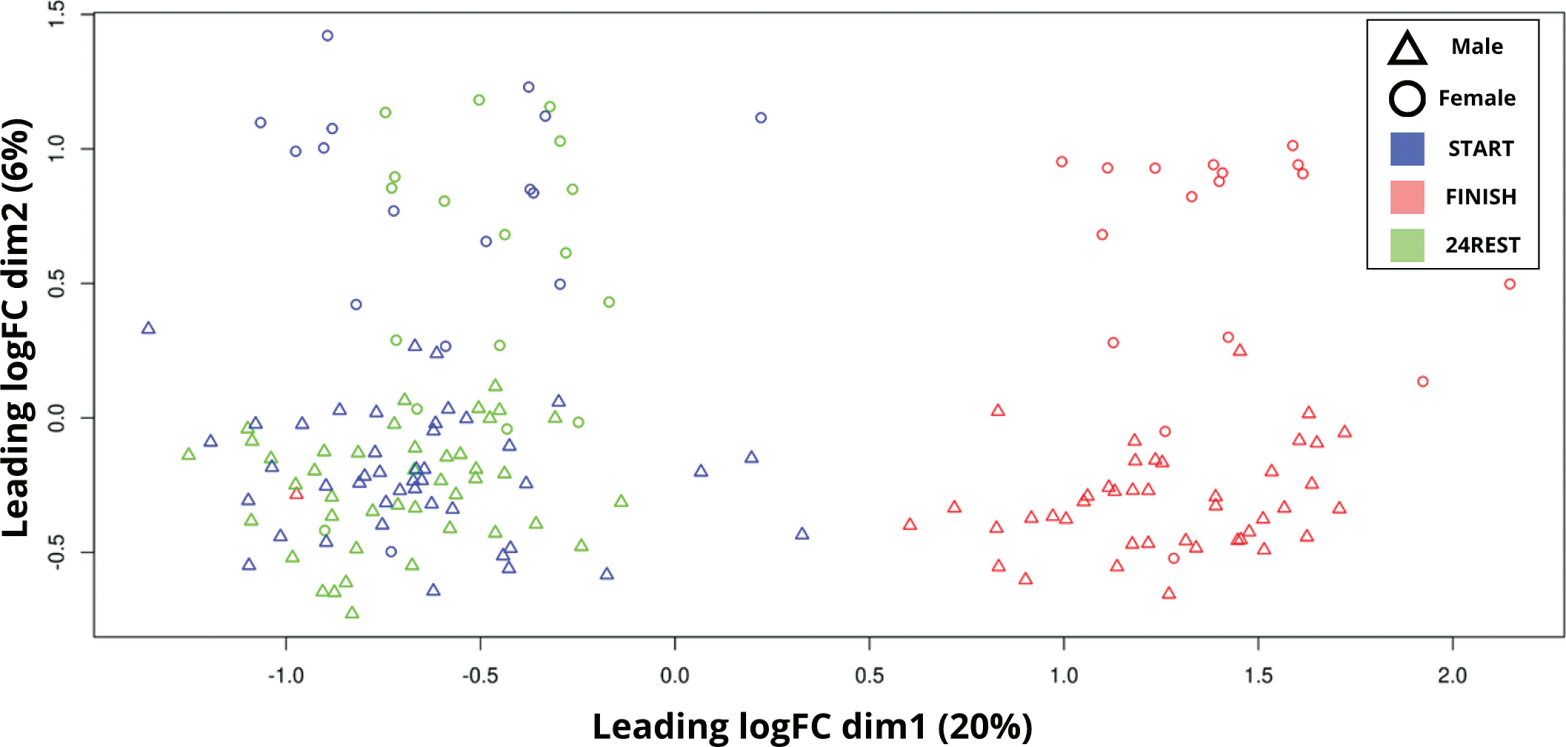
Principal component analysis (PCA) plot showing the clustering of samples based on transcriptomic profiles. Samples are colored by group: red for START, green for 24REST, and black for FINISH. Each point represents an individual sample, with “Δ” denoting male and “○” denoting female. The X-axis (leading logFC dim 1) explains 20% of the variation, while the Y-axis (leading logFC dim 2) explains 6%. Distinct clustering is observed for the FINISH group, while START and 24REST show more overlap.

To identify potential covariates that might influence the analysis, a multidimensional scaling analysis was conducted. This analysis revealed variations among samples based on normalized RNA counts, as illustrated in Figure 2.

In dim1 (X-axis), a distinct separation between the START and 24REST samples was evident in comparison to the FINISH samples. Regarding the covariates, sex was identified as a variable influencing inter-variability, given the clear difference in dim2 between females and males. Additionally, intra-variability among samples also requires consideration.

### Differential gene expression analysis (DEGs)

Differential gene expression analysis revealed that 9,861 of the initial 14,554 genes were differentially expressed (DE) in C1, 9,730 in C2, and only 279 in C3. Given how the groups were compared, the overexpression in groups C1 and C2 determines that expression was significantly higher after the race (i.e., after strenuous exercise) than when at rest. While the overexpression of gene levels in comparison C3 indicates there was significantly higher level of gene expression 24 hours after the race compared to baseline levels. Finally, a deeper analysis of the DEGs was also performed for each group comparison, with a particular emphasis on the biological function of the most significant genes.

The genes in this comparison with significantly different expression levels were mainly associated with immune cell markers, chemokines, and interleukins (Table 2). Regarding immune cell markers, *CD48, CD19, LRRC8C, LRRC8D* and *CLEC10A* genes were found to be downregulated.

**TABLE 2:** Differentially expressed genes related to the immune system, ROS environment, and mitochondria in the START vs FINISH comparison C1. Two methods were used, DESeq2 and Limma, and p-values were adjusted by Bonferroni in both cases.

Ensembl ID	HGNC symbol	log2FC (DESeq2)	Adjusted p-value (DESeq2)	log2FC (Limma)	Adjusted p-value (Limma)
ENSG00000115604	IL18R1	3.08	2.81 × 10^−175^	2.95	2.6 × 10^−54^
ENSG00000184557	SOCS3	3.34	1.04 × 10^−155^	3.28	3.2 × 10^−53^
ENSG00000115590	IL1R2	2.88	1.1 × 10^−152^	2.90	8.88 × 10^−53^
ENSG00000160712	IL6R	1.29	1.48 × 10^−116^	1.30	2.66 × 10^−49^
ENSG00000121807	CCR2	1.52	5.13 × 10^−94^	1.48	3.53 × 10^−45^
ENSG00000115594	IL1R1	2.06	9.49 × 10^−102^	2.08	4.94 × 10^−45^
ENSG00000008130	NADK	1.24	1.07 × 10^−81^	1.27	1.21 × 10^−44^
ENSG00000243646	IL10RB	1.15	2.71 × 10^−105^	1.15	1.3 × 10^−43^
ENSG00000008517	IL32	-1.61	1.44 × 10^−81^	-1.56	1.34 × 10^−43^
ENSG00000271503	CCL5	-1.75	8.66 × 10^−72^	-1.71	1.38 × 10^−42^
ENSG00000211445	GPX3	2.48	2.26 × 10^−67^	2.38	2 × 10^−40^
ENSG00000170458	CD14	1.58	1.9 × 10^−81^	1.56	3.5 × 10^−39^
ENSG00000163823	CCR1	1.48	6.66 × 10^−41^	1.44	5.56 × 10^−38^
ENSG00000177575	CD163	1.50	1.02 × 10^−56^	1.46	3.5 × 10^−35^
ENSG00000136689	IL1RN	1.18	1.36 × 10^−24^	1.30	9.81 × 10^−29^
ENSG00000132514	CLEC10A	-1.21	3.45 × 10^−31^	-1.22	5.8 × 10^−28^
ENSG00000112486	CCR6	-1.00	3.7 × 10^−43^	-1.05	2.4 × 10^−27^
ENSG00000077238	IL4R	1.12	6.01 × 10^−39^	1.08	4.22 × 10^−27^
ENSG00000120833	SOCS2	-1.11	1.58 × 10^−43^	-1.13	1.86 × 10^−26^
ENSG00000172890	NADSYN1	0.49	1.29 × 10^−42^	0.48	2.77 × 10^−26^
ENSG00000150782	IL18	1.09	1.68 × 10^−43^	1.06	3.5 × 10^−26^
ENSG00000125538	IL1B	1.10	2.2 × 10^−30^	1.08	2.04 × 10^−23^
ENSG00000160791	CCR5	-0.91	4.57 × 10^−19^	-0.96	3.97 × 10^−21^
ENSG00000091181	IL5RA	-1.37	5.77 × 10^−22^	-1.37	5.21 × 10^−21^
ENSG00000119772	DNMT3A	0.41	9.31 × 10^−28^	0.41	8.09 × 10^−21^
ENSG00000163600	ICOS	-0.83	1.23 × 10^−23^	-0.89	1.07 × 10^−19^
ENSG00000197272	IL27	1.87	3 × 10^−22^	1.49	3.92 × 10^−17^
ENSG00000171488	LRRC8C	-0.51	6.12 × 10^−21^	-0.53	9.85 × 10^−14^
ENSG00000183813	CCR4	-0.68	5.32 × 10^−14^	-0.74	3.61 × 10^−11^
ENSG00000177455	CD19	-0.56	2.12 × 10^−8^	-0.54	8.31 × 10^−11^
ENSG00000173585	CCR9	-1.00	2.6 × 10^−9^	-0.89	5.53 × 10^−8^
ENSG00000171492	LRRC8D	-0.38	4.91 × 10^−11^	-0.40	6.09 × 10^−7^
ENSG00000170677	SOCS6	0.70	1.04 × 10^−9^	0.61	2.78 × 10^−6^
ENSG00000117091	CD48	-0.31	1.07 × 10^−11^	-0.29	6.5 × 10^−5^
ENSG00000274211	SOCS7	-0.31	5.11 × 10^−9^	-0.32	1.45 × 10^−4^
ENSG00000110324	IL10RA	-0.23	1.94 × 10^−7^	-0.22	0.002163

As for the chemokines, most of the downregulated genes were expressed by T cell lymphocytes (*CCR4, CCR5, CCR6, CCR9*). These cells play an important role in immunity and have also been found to be altered in similar studies [24]. Conversely, not all the genes related to cytokines were downregulated. Important specific cytokine receptors for monocytes, such as CCR2 and CCR1, were found to be overexpressed besides CCL5 being downregulated. Moreover, some genes related to the family of suppressors of cytokine signaling proteins were also downregulated (*SOCS2*, and *SOCS7*), while others were overexpressed (*SOCS3* and *SOCS6*).

The last family of genes that stood out as DEGs was interleukins (IL). The most downregulated interleukins included *IL-32, IL-10RA, ICOS*, and *IL5RA*. Conversely, *IL1R1, IL-18, IL-10RB, IL6R, IL-4R*, and *IL27* were overexpressed in the FINISH group compared with the START group.

Table 2 also includes proteins that regulate oxidative stress, including the glutathione peroxidase family (*GPX7*) and iron/manganese superoxide dismutase (*SOD1*), which were also downregulated, while *GPX3,* another gene from the glutathione peroxidase family and *DNMT3A*, which is an important gene related to reducing the oxidative environment was overexpressed.

Subsequently, several differentially expressed genes (DEGs) associated with inflammatory markers, as reported in previous studies were identified [25]. Notably, 21 out of 23 previously documented inflammatory markers were found to be significantly differentially expressed (Table 3).

**TABLE 3:** Differentially expressed genes related to inflammatory markers in the START vs FINISH comparison C1. Two methods were used, DESeq2 and Limma, and p-values were adjusted by Bonferroni in both cases.

Ensembl ID	HGNC symbol	log2FC (DESeq2)	Adjusted p-value (DESeq2)	log2FC (Limma)	Adjusted p-value (Limma)
ENSG00000136869	TLR4	2.08	2.13 × 10^−133^	2.10	2.58 × 10^−54^
ENSG00000100985	MMP9	3.57	2.93 × 10^−116^	3.66	4.07 × 10^−51^
ENSG00000160712	IL6R	1.29	1.48 × 10^−116^	1.30	2.66 × 10^−49^
ENSG00000159128	IFNGR2	1.24	2.09 × 10^−99^	1.27	5.8 × 10^−46^
ENSG00000161921	CXCL16	1.68	6.07 × 10^−96^	1.70	2.48 × 10^−45^
ENSG00000173110	HSPA6	1.72	2.36 × 10^−102^	1.73	2.61 × 10^−45^
ENSG00000121807	CCR2	1.52	5.13 × 10^−94^	1.48	3.53 × 10^−45^
ENSG00000115594	IL1R1	2.06	9.49 × 10^−102^	2.08	4.94 × 10^−45^
ENSG00000243646	IL10RB	1.15	2.71 × 10^−105^	1.15	1.3 × 10^−43^
ENSG00000271503	CCL5	-1.75	8.66 × 10^−72^	-1.71	1.38 × 10^−42^
ENSG00000137462	TLR2	1.57	1.83 × 10^−86^	1.61	8 × 10^−42^
ENSG00000107485	GATA3	-1.19	2.44 × 10^−70^	-1.21	1.25 × 10^−35^
ENSG00000069702	TGFBR3	-1.68	3.26 × 10^−58^	-1.72	4.67 × 10^−33^
ENSG00000136689	IL1RN	1.18	1.36 × 10^−24^	1.30	9.81 × 10^−29^
ENSG00000077238	IL4R	1.12	6.01 × 10^−39^	1.08	4.22 × 10^−27^
ENSG00000116157	GPX7	-1.10	2.37 × 10^−47^	-1.12	2.85 × 10^−24^
ENSG00000125538	IL1B	1.10	2.2 × 10^−30^	1.08	2.04 × 10^−23^
ENSG00000135966	TGFBRAP1	-0.53	1.02 × 10^−30^	-0.55	6.71 × 10^−19^
ENSG00000232810	TNF	-0.61	1.09 × 10^−17^	-0.60	9.4 × 10^−15^
ENSG00000183813	CCR4	-0.68	5.32 × 10^−14^	-0.74	3.61 × 10^−11^
ENSG00000142168	SOD1	-0.49	1.43 × 10^−13^	-0.45	4.27 × 10^−6^

The results of the C2 comparison were very similar to those of C1. All the genes reported in C1 were also differentially expressed in C2.

As previously stated, only 279 genes were differentially expressed in C3, representing less than 5% of the differentially expressed (DE) genes identified in the other two comparisons. In C3 (Table 4), genes related to endocytosis, and vesicle biology such as *APP, VPS13D,* and *LRP1* were overexpressed, whereas *TMEM179B, EMP3,* and *COPE* were downregulated. Meanwhile, mitochondrial function and metabolism genes, including *NDUFS7, PHB2,* and *ALKBH7*, were downregulated. On the other hand, RNA processing, and ribosomal components, such as *EIF3A, PRRC2C,* and *YLPM1* were overexpressed, while *RPL28, GET3,* and *CARHSP1* were downregulated. Finally, transcriptional regulation and chromatin remodeling genes *ARID1A* and *AHNAK* were overexpressed, whereas *ZNF581* was downregulated.

**TABLE 4:** The top 10 differentially expressed genes in START vs 24REST comparison (C3). Two methods were used, DESeq2 and Limma, and p-values were adjusted by Bonferroni in both cases.

Ensembl ID	HGNC symbol	log2FC (DESeq2)	Adjusted p-value (DESeq2)	log2FC (Limma)	Adjusted p-value (Limma)
ENSG00000142192	APP	0.38	1.66 × 10^−7^	0.40	9.45 × 10^−9^
ENSG00000185475	TMEM179B	-0.33	3.02 × 10^−8^	-0.31	9.13 × 10^−8^
ENSG00000124942	AHNAK	0.36	9.35 × 10^−7^	0.39	1.22 × 10^−7^
ENSG00000134250	NOTCH2	0.40	1.4 × 10^−6^	0.41	2.56 × 10^−7^
ENSG00000048707	VPS13D	0.23	7.45 × 10^−8^	0.24	9.78 × 10^−7^
ENSG00000108107	RPL28	-0.47	1.21 × 10^−7^	-0.42	2.35 × 10^−6^
ENSG00000117523	PRRC2C	0.31	1.57 × 10^−6^	0.33	2.85 × 10^−6^
ENSG00000119596	YLPM1	0.26	4.1 × 10^−7^	0.28	5.53 × 10^−6^
ENSG00000240972	MIF	-0.45	3.02 × 10^−8^	-0.41	5.62 × 10^−6^
ENSG00000115286	NDUFS7	-0.36	1.66 × 10^−7^	-0.33	6.73 × 10^−6^
ENSG00000117713	ARID1A	0.29	7.74 × 10^−6^	0.32	9.53 × 10^−6^
ENSG00000198356	GET3	-0.26	1.8 × 10^−7^	-0.23	1.11 × 10^−5^
ENSG00000215021	PHB2	-0.24	3.73 × 10^−7^	-0.21	1.49 × 10^−5^
ENSG00000171425	ZNF581	-0.35	1.36 × 10^−6^	-0.33	1.66 × 10^−5^
ENSG00000125652	ALKBH7	-0.44	4.24 × 10^−7^	-0.41	1.98 × 10^−5^
ENSG00000153048	CARHSP1	-0.33	3.38 × 10^−5^	-0.32	2.43 × 10^−5^
ENSG00000107581	EIF3A	0.33	3.02 × 10^−8^	0.34	2.57 × 10^−5^
ENSG00000123384	LRP1	0.38	4.4 × 10^−5^	0.41	2.66 × 10^−5^
ENSG00000142227	EMP3	-0.39	5.2 × 10^−6^	-0.36	3.23 × 10^−5^
ENSG00000105669	COPE	-0.31	3.26 × 10^−6^	-0.29	3.92 × 10^−5^

### Likelihood Ratio Test (LRT) and clusterization between groups

**FIG. 3:**
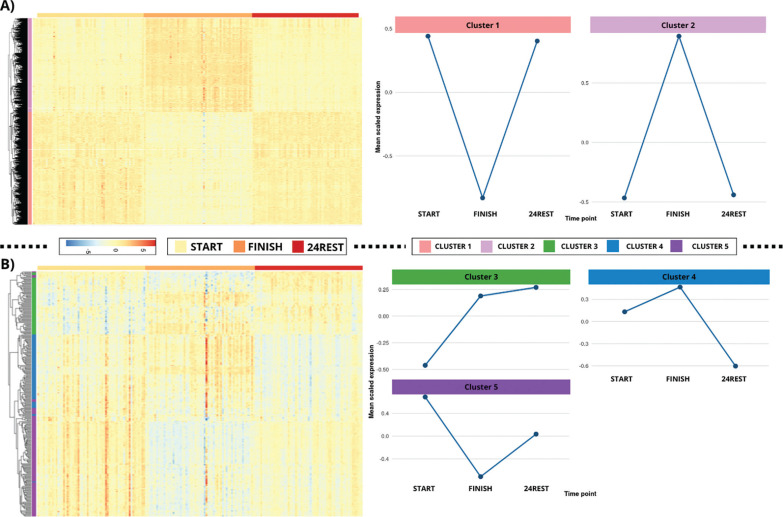
Hierarchical clustering and k-means analysis of gene expression trajectories. Gene expression trajectories were explored using k-means clustering, with the optimal number of clusters determined via the elbow method. Figure 3A shows two predominant clusters: a first group with increased expression immediately post-marathon that returned to baseline after 24 hours, and a second group exhibiting decreased expression post-marathon followed by recovery at 24 hours. Figure 3B highlights a smaller subset of genes (279 genes) displaying three distinct temporal patterns, from differential expression genes 24 hours after the race compared to baseline.

Following the likelihood ratio test, 16,401 significant genes were identified and used to determine clusters of similar expression patterns. The elbow method indicated the presence of two major clusters, and k-means clustering was subsequently applied with this specification. A heatmap incorporating hierarchical clustering via the complete linkage method was generated to visualize these clusters. The results revealed two distinct clustering of FINISH samples, whereas START and 24REST samples exhibited more similar expression profiles, indicating substantial transcriptomic alterations immediately after the marathon. Two primary patterns were observed: one group of genes upregulated at FINISH and another downregulated at FINISH, both returning toward baseline levels at 24REST (Figure 3a).

To examine finer subclusters, the analysis was repeated for the 279 genes identified in pairwise comparison 3. In this case, the elbow method suggested three clusters, which were visualized (y-axis) in a heatmap ordered by time group along the x-axis (Figure 3b). The first cluster (cluster 3) showed increased expression at FINISH, further rising at 24REST. The second cluster (cluster 4) exhibited elevated expression at FINISH compared with START, followed by a pronounced decrease at 24REST, falling below baseline levels. The third cluster (cluster 5) displayed decreased expression at FINISH relative to START, with partial recovery at 24REST that did not reach baseline levels.

**FIG. 4:**
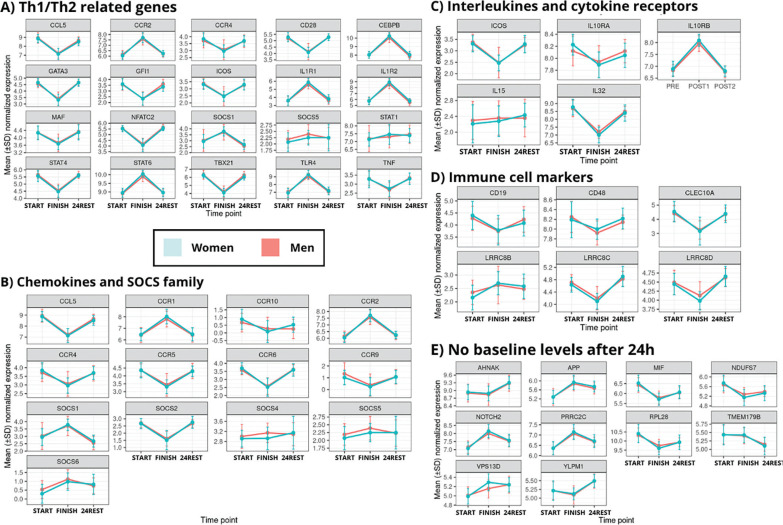
Temporal expression patterns of representative gene subsets across immune pathways. This figure comprises five subpanels (a–e), illustrating the temporal variation in representative genes across key functional categories: immune markers, chemokines/SOCS, interleukins, Th1/Th2 signaling, and genes with no baseline-level expression at 24 hours.

Consistently, visualization of C1 gene expression stratified by sex (Figure 4) reproduced the five cluster patterns observed in the overall dataset.

Finally, to evaluate potential sex-specific transcriptional responses, an interaction model including sex and group was tested using DESeq2 while adjusting for age and batch effects. After bonferroni correction, no genes showed statistically significant interaction effects, indicating that the overall temporal expression changes following the marathon were similar in both men and women

Meanwhile, when comparing potential performance-specific transcriptional responses, the interaction between the model including finishing time and group display IRAK4 (ENSG00000198001) as the only single differentially expressed gene, with a bonferroni adjusted p-value of 0.04 but with a very low logFC -0.0013.

### Gene ontology term and KEGG pathway enrichment analyses

**FIG. 5:**
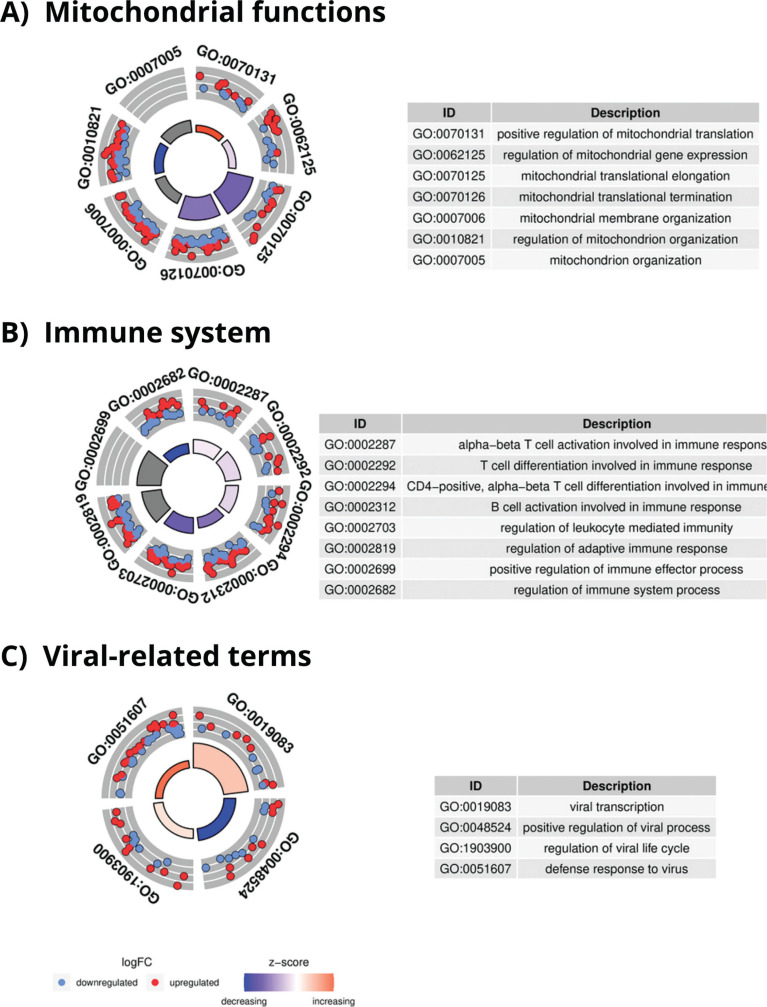
GO enrichment analysis results in C2. A) Enriched GO terms related to mitochondrial functions in FINISH vs 24REST conditions with an adjusted p-value Bonferroni of < 0.05. B) Enriched GO terms related to immune system in FINISH vs 24REST conditions with an adjusted p-value Bonferroni of < 0.05. C) Enriched GO terms related to viruses (c) in FINISH vs 24REST conditions with an adjusted p-value Bonferroni of < 0.05.

After performing conditional GO analysis and filtering the results by size and counts, 310 enriched GO terms were found in C1, 318 in C2, and 43 in C3. A complete list of all GO terms is given in Tables S6 for C1 and S7 for C2. C1 and C2 returned similar results, as previously reported in the DEGs analysis. The top enriched GO terms for C2, related to mitochondrial functions (Figure 5a) and T and B cell activation (Figure 5b) were mainly downregulated while virusrelated (Figure 5c) GO terms were upregulated.

Finally, results obtained in C3 (Table S8) show that most of the enriched GO terms were related to energy generation processes, such as the electron transport chain, several GO terms related with the ATP synthesis and the mitochondrial respiratory chain complex I assembly.

On the other hand, the total number of KEGG pathways enriched were 58, 57, and 12 for comparisons C1, C2, and C3, respectively. Tables S9 and S10 show the total list of pathways for comparisons C1 and C2, while the list of enriched pathways for C3 is shown in Table 5.

**TABLE 5:** Enriched KEGG pathways in the START vs 24REST comparison (C3). All p-values were adjusted by Bonferroni method.

Category	Subcategory	KEGG ID	Pathway	Count	Gene ratio
Human Diseases	Neurodegenerative disease	hsa05016	Huntington disease	27	27/144

Organismal Systems	Environmental adaptation	hsa04714	Thermogenesis	23	23/144

Metabolism	Energy metabolism	hsa00190	Oxidative phosphorylation	17	17/144

Human Diseases	Neurodegenerative disease	hsa05014	Amyotrophic lateral sclerosis	26	26/144

Human Diseases	Neurodegenerative disease	hsa05012	Parkinson disease	22	22/144

Human Diseases	Neurodegenerative disease	hsa05020	Prion disease	22	22/144

Human Diseases	Cardiovascular disease	hsa05415	Diabetic cardiomyopathy	19	19/144

Human Diseases	Neurodegenerative disease	hsa05010	Alzheimer disease	26	26/144

Human Diseases	Cancer: overview	hsa05208	Chemical carcinogenesis – reactive oxygen species	19	19/144

Human Diseases	Neurodegenerative disease	hsa05022	Pathways of neurodegeneration – multiple diseases	27	27/144

Genetic Information Processing	Translation	hsa03010	Ribosome	13	13/144

Human Diseases	Endocrine and metabolic disease	hsa04932	Non-alcoholic fatty liver disease	11	11/144

**Table:** 

Category	Background ratio	Rich factor	Fold enrichment	z-score	p-value	Adjusted p-value	q-value
Human Diseases	306/8106	0.088	4.97	9.51	2.9 × 10^−12^	5.95 × 10^−10^	5.65 × 10^−10^

Organismal Systems	232/8106	0.099	5.58	9.52	1.34 × 10^−11^	2.75 × 10^−9^	1.3 × 10^−9^

Metabolism	134/8106	0.127	7.14	9.64	1.62 × 10^−10^	3.33 × 10^−8^	1.05 × 10^−8^

Human Diseases	364/8106	0.071	4.02	7.93	8.59 × 10^−10^	1.76 × 10^−7^	4.18 × 10^−8^

Human Diseases	266/8106	0.083	4.66	8.15	1.32 × 10^−9^	2.7 × 10^−7^	5.13 × 10^−8^

Human Diseases	273/8106	0.081	4.54	7.99	2.15 × 10^−9^	4.41 × 10^−7^	6.58 × 10^−8^

Human Diseases	203/8106	0.094	5.27	8.28	2.52 × 10^−9^	5.16 × 10^−7^	6.58 × 10^−8^

Human Diseases	384/8106	0.068	3.81	7.59	2.7 × 10^−9^	5.54 × 10^−7^	6.58 × 10^−8^

Human Diseases	223/8106	0.085	4.80	7.73	1.2 × 10^−8^	2.47 × 10^−6^	2.6 × 10^−7^

Human Diseases	476/8106	0.057	3.19	6.63	5.68 × 10^−8^	1.16 × 10^−5^	1.11 × 10^−6^

Genetic Information Processing	158/8106	0.082	4.63	6.20	4.15 × 10^−6^	8.51 × 10^−4^	7.35 × 10^−5^

Human Diseases	155/8106	0.071	3.99	5.06	9.21 × 10^−5^	0.018878	0.001494

The pathway analysis results closely resembled those of the GO term analysis, strengthening the significance of the findings. Moreover, the results of the C1 and C2 comparisons were also similar to each other but differed from C3. For comparisons C1 and C2, the pathways related to apoptosis, cellular senescence, mitophagy, and necroptosis were enriched. Further top enriched terms were a group of pathways related to lipid metabolism, including fatty acid metabolism, lipid and atherosclerosis, and some sphingolipid signaling pathways. In C2, insulin resistance and vascular endothelial growth factor (VEGF) signaling pathways were enriched too.

Gene expression trajectories were explored using k-means clustering, with the optimal number of clusters determined via the elbow method. Figure 3A shows two predominant clusters: a first group with increased expression immediately post-marathon that returned to baseline after 24 hours, and a second group exhibiting decreased expression post-marathon followed by recovery at 24 hours. Figure 3B highlights a smaller subset of genes (279 genes) displaying three distinct temporal patterns, from differential expression genes 24 hours after the race compared to baseline.

Finally, in C3, those pathways related to oxidative environment maintenance were among the top significant results (Table 5).

## DISCUSSION

Previous studies have investigated the effect of strenuous exercise on human gene expression. The present study was carried out in non-elite athletes to measure the effects of an endurance event such as running a marathon and determine if gene expression levels recovered after 24 hours. The first significant finding was that 60% of the genome was differentially expressed immediately after finishing the marathon compared to baseline levels, indicating that athletes experience major transcriptomic alteration due to endurance exercise. Moreover, 279 genes remained differentially expressed 24 hours after the marathon, suggesting that subjects had not recovered their baseline gene expression levels even after 24 hours of rest.

In general, the results reflect that strenuous exercise can induce an inflammatory response, activate an oxidative environment, and downregulate the immune system. Previous studies have reported that endurance exercise induces an inflammatory environment, which indicates that long periods of strenuous exercise can generally lead to higher levels of inflammatory mediators and therefore may increase the risk of injury and chronic inflammation [26]. The results obtained in this study mirrored these findings for 21 out of 23 inflammatory markers that were significantly differentially expressed at C1 (Table 3).

### Immune repression with monocyte activation after marathon running

This figure comprises five subpanels (a–e), illustrating the temporal variation in representative genes across key functional categories: immune markers, chemokines/SOCS, interleukins, Th1/Th2 signaling, and genes with no baseline-level expression at 24 hours.

Subsequently, most immune-related genes were downregulated after the marathon. In Condition 1 (C1), markers involved in the B cell life cycle, specifically those associated with B lymphocyte activation (CD48), pre-B cells (CD19), and LRRC8C and LRRC8D, which are essential for B cell maturation and belong to the T cell activation leucine-rich repeat protein family, were downregulated. Similarly, chemokine receptors expressed by T lymphocytes (CCR4, CCR5, CCR6, and CCR9) showed reduced expression (Table 2). Within the suppressor of the cytokine signaling family, SOCS2 and SOCS7 were downregulated, whereas SOCS3 and SOCS6 were upregulated. In addition, CCL5, a chemokine that mediates chemotaxis for T cells, eosinophils, and basophils and promotes proliferation and activation of natural killer cells, was also downregulated [27].

Furthermore, several interleukins were suppressed in C1, including IL5 and IL32. IL32, a proinflammatory cytokine, contributes to the pathogenesis of autoimmune diseases such as rheumatoid arthritis, while also conferring protection against certain respiratory infections, including tuberculosis [28–29]. Moreover, IL5 regulates genes associated with B cell terminal maturation, as the markers commented above [30].

However, not all immune-related genes were downregulated. The IL6 receptor, a cytokine produced by myocytes during muscle contraction, and genes related to IL10 were significantly overexpressed [31]. This overexpression was not consistent across all IL10-related genes. While ICOS, a protein enhancing IL10 synthesis, and IL10RB were downregulated, IL10RA was upregulated in C1.

Among the overexpressed immune genes, most were specific to monocytes. These included CD14, a surface antigen predominantly expressed on monocytes; CD163, identified as a potential biomarker of monocyte activation in ischemic stroke; and CLEC10A, a gene involved in inflammatory and immune responses (Table 2) [32]. Although CLEC10A mRNA expression occurs in intermediate monocytes, its predominant expression is found in dendritic cells. Additionally, key monocyte chemokine receptors, including CCR2 and CCR1, were overexpressed [33]. IL18 and its receptor IL18R1 were overexpressed, consistent with IL18’s role in enhancing monocyte activation [34]. IL1B, a cytokine primarily produced by monocytes and a key early proinflammatory mediator, was also upregulated, along with its antagonist receptor IL1RN and receptors IL1R1 and IL1R2 [35].

Despite the widespread downregulation observed in other immune components, the upregulation of monocyte markers, specific cytokines, and interleukins indicates that monocyte activation persists following strenuous exercise. Previous studies have shown that intense physical exertion activates monocytes, leading to acute inflammation and hypoxemia. These findings align with the present results, underscoring the pivotal role of monocytes in the immune response to marathon running [36].

Following prolonged exercise, several genes regulating oxidative stress, including the glutathione peroxidase family member GPX7 and superoxide dismutase SOD1, were downregulated. This oxidative environment may represent the molecular link between monocyte chemotaxis and inflammatory pain [37]. Further studies are required to clarify the role and significance of monocytes in this context. Conversely, DNMT3A was overexpressed in C1, consistent with reports showing increased expression in oxidative skeletal muscle after endurance exercise [38]. DNMT3A deficiency is associated with excessive production of reactive oxygen species, a key contributor to muscle dysfunction.

### Neuroprotective, chromatin remodeling and mitochondrial adaptations 24 Hours after marathon running

In C3, APP and LRP1 were found overexpressed compared with baseline levels. Both genes are associated with Alzheimer’s disease, and their upregulation following exercise has been reported to exert neuroprotective effects [39]. In particular, LRP1 expression typically declines with disease progression, whereas its enhancement through exercise or pharmacological approaches has been linked to improved amyloid-beta clearance and attenuation of neuroinflammatory responses [40].

EIF3A, a subunit of the eukaryotic translation initiation factor 3 complex, was also overexpressed, suggesting increased regulation of protein synthesis and potential involvement in the cellular response to DNA damage [41]. In contrast, genes related to mitochondrial function, including NDUFS7, PHB2, and ALKBH7, were downregulated, indicating a transient reduction in mitochondrial activity during recovery.

Additionally, the overexpression of ARID1A and AHNAK points to transcriptional and chromatin remodeling responses associated with exercise-induced histone acetylation. Such epigenetic modulation promotes chromatin decompaction and activation of exercise-responsive genes, reflecting ongoing molecular adaptation during the postexercise recovery phase [42].

### Immune recovery but persistent mitochondrial alterations 24 Hours after marathon running

The enrichment analysis showed that downregulated genes were primarily associated with Gene Ontology (GO) terms and KEGG pathways related to B and T lymphocytes and other immune system components. This immune suppression may contribute to the increased susceptibility to infection following endurance exercise, as previously reported [43, 44]. These findings are consistent with evidence that endurance athletes experience marked physiological stress during prolonged exercise, leading to transient immunodepression and a higher risk of upper respiratory tract infections due to immune weakening [45]. Conversely, in C3, the results indicated that 24 hours after exercise, immune-related pathways had largely returned to baseline levels, suggesting recovery of immune function during this period.

In C2, enrichment of KEGG pathways related to diabetic cardiomyopathy and atherosclerosis was observed. Previous studies have reported a higher prevalence of coronary artery calcification among long-term marathon athletes, particularly males, which may reflect exercise-induced cardiovascular remodeling [46].

At C3, enriched GO terms and KEGG pathways were associated with mitochondrial activity, energy metabolism, and oxidative stress. Specifically, pathways such as ATP synthesis coupled electron transport, ATP metabolic process, mitochondrial electron transport from NADH to ubiquinone, and oxidative phosphorylation were significantly enriched. These findings indicate that non-elite athletes exhibited reduced mitochondrial energy generation capacity 24 hours after the race compared with their baseline condition.

### Identification of two clusters of recovered genes and three clusters of differentially expressed genes 24 hours after marathon completion

Following the enrichment analysis, global transcriptomic profiling revealed two primary gene expression patterns across the three conditions. Genes that returned to baseline 24 hours after the marathon were assigned to one of these clusters, reflecting overall recovery dynamics. Examination of the 279 genes differentially expressed in C3 identified three subclusters with distinct temporal behaviors. Cluster 3, which included APP and LRP1, displayed continued elevation at 24 hours, suggesting prolonged post-exercise activation. Cluster 4 showed upregulation immediately after the marathon, followed by a pronounced decline at 24 hours to levels below baseline. Cluster 5 comprised genes downregulated at the finish line that partially recovered at 24 hours but did not reach baseline levels. These patterns illustrate heterogeneous recovery kinetics among specific genes, highlighting persistent transcriptomic alterations in the early post-exercise period.

Although principal component analysis (PCA) demonstrated clear separation between men and women at the global transcriptomic level (Figure 2), reflecting expected baseline biological differences, our formal interaction analysis did not identify statistically significant sex-dependent transcriptional responses to the marathon after multiple-testing correction. This likely reflects, at least in part, the unbalanced sample composition (42 men and 18 women), which limits power to detect smaller sex effects. The inclusion of sex as a covariate in our principal models mitigates potential confounding, but the present dataset may still underrepresent female-specific molecular signatures. Future studies with larger and more balanced cohorts are warranted to better characterize potential sex-dependent regulatory responses to endurance exercise.

In conclusion, marathon running induces widespread transcriptomic changes, with 9,874 of 14,554 detected genes differentially expressed immediately after the race. We observed immune repression with increased monocyte activity, alongside changes in oxidative stress and inflammatory markers. At 24 hours post-marathon, immune gene expression largely returned to baseline, while mitochondrial function, energy metabolism, and chromatin regulation remained altered. Notably, two genes associated with Alzheimer’s disease, APP and LRP1, remained overexpressed at 24 hours, a pattern previously linked to neuroprotective effects associated with exercise. Clustering analysis identified two groups of recovered genes and three groups of genes with sustained changes, reflecting distinct temporal patterns of recovery.

These results provide a detailed view of transcriptional responses to endurance exercise in non-elite athletes. They highlight gene expression patterns that recover rapidly their baseline expression after endurance exercise and those that remain altered after 24 hours, offering data that could guide personalized training, nutritional strategies, and recovery planning. This study contributes to understanding the temporal dynamics of gene expression following prolonged endurance exercise and identifies targets for future longitudinal studies.

### Limitations

While this study provides robust transcriptomic insights into marathon-induced responses, some limitations should be noted. Biochemical parameters such as CRP, creatine kinase, and lactate were not collected due to the practical challenges of multiple blood draws during the event. The dataset was adjusted to account for gender imbalance, though some residual heterogeneity may remain given the higher proportion of men in the cohort. Additionally, key findings were not independently validated by RT-qPCR, which could further support the results. Future studies combining biochemical measures, larger cohorts, and independent validation would further strengthen these observations.

## Data Availability

The raw data and some datasets generated and/or analyzed during the course of the study are not publicly available due to confidentiality and privacy considerations. However, interested parties may request access to the data by submitting a reasonable request to the corresponding author of the article. All requests will be subject to review by the Ethical Committee of Hospital Sant Pau to ensure compliance with ethical guidelines and patient privacy protections. Approval from the committee is required before any data can be shared. Requests for access can be sent directly to the corresponding author, who will facilitate the review process with the Ethical Committee of Hospital Sant Pau.

## Supplementary materials

Supplementary tables are available at the following LINK
